# 

**Published:** 1918-08-03

**Authors:** 


RATES OF SUBSCRIPTION:

Twelve months, 10/10; Six months, 5/5; Three months, 2/9, post free to any address in the United Kingdom. Abroad, Twelve months, 13/-; Six months, 6/6; Three months, 3/3, post free. **THE HOSPITAL "Index" to Volume LXI., October 19 7 to March 1918 is now ready, price 3½d. per copy, post free.** Address The Manager, THE HOSPITAL, "The Hospital" Building, 28/29 Southampton Street, Strand, London, W.C. 2.

### Recent Official Publications.

(To be obtained from H.M. Stationery Office, Imperial House, Kingsway, W.C. 2. All prices quoted are postage free.)

Midwives Bill. As amended on Report. 3d.

Public Health. Scotland. Prevention of Epidemic, Endemic, or Infectious Diseases. Public Health (Tuberculosis) Regulations (Scotland), July 8, 1918. 1½d.

The Nomenclature of Diseases, drawn up by a Joint Committee appointed by the Royal College of Physicians of London.

### Beth Israel Hospital, New York.

The American War and Navy Department have received a munificent offer from the Jewish Congressman, Mr. Isaac Siegel, on behalf of Beth Israel Hospital, New York, to erect in New York, for either department, a hospital with 500 beds, costing £250,000.

### Matrons' and Sisters' Appointments.

Ayr County Hospital.—Miss I. M. Crichton has been appointed matron. She was trained at the Chalmers Hospital, Edinburgh, and has since been charge nurse at the Victoria Infirmary, Glasgow, and theatre, x-ray, and housekeeping sister, and assistant matron at the Chalmers Hospital.

Bethnal Green Military Hospital.—Miss D. V. Barclay has been appointed ward sister. She was trained at Edmonton Infirmary, and has since been sister at the West Kent General Hospital and staff nurse at Edmonton Military Hospital.

Cheltenham General Hospital.—Miss L. C. Fox-Dewies has been appointed matron. She was trained at Birmingham General Hospital, and has since been ward sister, theatre and out-patients' sister, night sister, and home sister at Cheltenham General Hospital.

Kensington Infirmary, Marloes Road.—Miss Ella Jones has been appointed superintendent of night nurses. She was trained at Kensington Infirmary, where she has since been staff nurse, midwifery pupil, and ward sister.

Leeds Maternity Hospital.—Mrs. Marian Danes has been appointed home sister. She was trained at Derbyshire Royal Infirmary, and has since been night sister at Dorset County Hospital, home sister at a private nursing home, nurse-matron of Dorset Borough Hospital, and matron of Calverley Joint Hospital, Bradford.

Liverpool Home of Recovery, Allerton Tower.—Miss Grace C. Maywood has been appointed matron. She was trained at Manchester Royal Infirmary, has since been assistant matron of the Red Cross Hospital, Netley, of a private nursing home at Ilfracombe, and has done private nursing in London previous to the war.

Montrose Infectious Diseases Hospital.—Miss A. R. Hay has been appointed nurse-matron. She was trained at Alloa Infectious Diseases Hospital and at the Royal Infirmary, Halifax.

Perth Sanatorium, Hillside House.—Miss A. N. Blair has been appointed sister. She was trained at Glasgow Royal Infirmary, and has since been sister at the Royal Hospital for Incurables, West Hill, Putney, at the Broomhill Hospital for Incurables, Kirkintilloch, at the Consumption Sanatoria of Scotland, Bridge of Weir, and matron of the Yorkshire Home at Harrogate.

Royal Albert Edward Infirmary and Dispensary, Wigan.—Miss Henrietta Follevaag has been appointed theatre sister and Miss S. A. Eddy ward sister. Miss Follevaag was trained at the Royal Albert Edward Infirmary and Miss Eddy at Sheffield Royal Infirmary.

St. Nicholas Home Hospital for Crippled Children, Pyrford, Woking.—Miss M. A. Mann has been appointed head matron. She was trained at Preston and the County of Lancashire Royal Infirmary, and has since been sister at St. Mary's Infirmary, Islington; assistant matron of Gloucester County Hospital; and matron of Cossham Hospital, Bristol, Bradford Incorporated Nurses' Institute, and the North Devon General Infirmary, Barnstaple.

**Note.—Particulars of Appointments for insertion in this column will be welcomed.**

### Health of Troops in Mesopotamia.

Mr. Macpherson informed Sir J. Jardine that he was glad to state that the hot-weather conditions in Mesopotamia are much less trying this year, and that the health of the troops shows marked improvement since last year. The provision of medical officers and hospitals is adequate, and there is an ample supply of vegetables, fruit, and comforts.

**☛ Owing to pressure on space "The Institutional Worker" is unavoidably held over.**

